# Potential usefulness of C-reactive protein and procalcitonin determination in patients admitted for neurological disorders in rural Democratic Republic of Congo

**DOI:** 10.1038/s41598-019-51925-z

**Published:** 2019-10-29

**Authors:** Emmanuel Bottieau, Deby Mukendi, Jean-Roger Lilo Kalo, Pascal Lutumba, Barbara Barbé, Kadrie Ramadan, Marjan Van Esbroeck, Jan Jacobs, Cedric P. Yansouni, François Chappuis, Marleen Boelaert, Andrea S. Winkler, Kristien Verdonck

**Affiliations:** 1 Department of Clinical Sciences, Institute of Tropical Medicine, Antwerp, Belgium; 2 Institut National de Recherche Biomédicale, Kinshasa, Democratic Republic of Congo; 3 Université de Kinshasa, Kinshasa, Democratic Republic of Congo; 4 Department of Microbiology and Immunology, KU Leuven, Leuven, Belgium; 5 JD MacLean Centre for Tropical Diseases, McGill University Health Centre, Montreal, Canada; 6 Division of Tropical and Humanitarian Medicine, Geneva University Hospitals and University of Geneva, Geneva, Switzerland; 7 Department of Public Health, Institute of Tropical Medicine, Antwerp, Belgium; 8 Department of Neurology, Technical University of Munich, Munich, Germany; 9 Centre for Global Health, University of Oslo, Oslo, Norway

**Keywords:** Biomarkers, Neurology

## Abstract

In low-resource hospitals of central Africa, neurological disorders are frequent and etiologies very diverse. The difficulty to identify invasive bacterial infections in this setting results in major antibiotic overuse. Biomarkers such as C-reactive protein (CRP) and procalcitonin (PCT) may help discriminate these conditions. We retrospectively determined the concentrations of CRP and PCT in the sera of patients consecutively enrolled from 2012 to 2015 in an etiological study on neurological disorders at the rural hospital of Mosango, Democratic Republic of Congo. Invasive bacterial infection had been diagnosed by the demonstration of a bacterial pathogen in cerebrospinal fluid or blood cultures or the presence of radiological pneumonia. Sera of 313 (89.2%) and 317 (90.3%) of the 351 enrolled participants were available for determination of CRP and PCT concentrations respectively. Areas under the receiver operating characteristic curves for invasive bacterial infection, diagnosed in 19 tested cases, were 94.3% for CRP and 91.7% for PCT. No single case had a normal CRP concentration (<10 mg/L). Our data, although limited, suggest that CRP or PCT concentrations may help discriminate invasive bacterial infections in patients with neurological disorders in tropical settings and that normal CRP values could assist in withholding antibiotics.

## Introduction

Neurological disorders represent a major burden for health systems of both high- and low-income countries. They may be caused by a wide variety of infectious and non-infectious etiologies, but the precise epidemiological spectrum is relatively unknown in settings with limited diagnostic facilities, such as in Central African rural hospitals^1^.

In the group of infectious etiologies that may present with neurological symptoms, invasive bacterial infections such as bacterial meningitis, bacteremia and severe sepsis should be detected with the highest priority because early antibiotic administration improves outcomes^2^. Antibiotics are however of no utility for the non-bacterial and non-infectious causes of neurological disorders, and their indiscriminate use may, on the one hand, undertreat some severe but curable non-bacterial infections while, on the other hand, fuel antimicrobial resistance. In settings with limited facilities such as in most rural Africa, first-line health care workers may feel overwhelmed by severely ill patients with neurological disorders. Clinical experience suggests that empirical treatment with antibiotics is nearly universal once malaria has been ruled out, as has been repeatedly observed for febrile illnesses^3^.

In high-income settings, two markers of inflammation and sepsis, C-reactive protein (CRP) and procalcitonin (PCT), are widely used to help discriminate infectious/inflammatory diseases from other conditions and to distinguish bacterial from viral infections^4,5^. Depending on the clinical scenario and setting in which they have been evaluated, both biomarkers have variable but similar overall performance^6^. Several studies have shown that the use of these tools can lead to a sizeable reduction in antibiotic overuse and even improves survival of adult populations in primary or intensive care settings^7–9^. Nevertheless, some controversy persists^10,11^. In clinical practice, it is common to use two cutoff concentrations for biomarkers: a lower value under which bacterial infection can be safely ruled out and a higher value above which it is usually ruled in^6,12^. Concentrations between these two cutoffs often constitute a grey zone for management.

The diagnostic value of both biomarkers is also increasingly explored in various low-resource settings, for different clinical syndromes, and at different cutoffs. Several point-of-care immunochromatography-based qualitative and semi-quantitative Rapid Diagnostic Tests (RDTs) have been developed for CRP and PCT determination, and some commercially available CRP RDTs have been validated in tropical settings^13^. Attention has mainly focused on the diagnostic value of those biomarkers in respiratory tract infections in sub-Saharan Africa^14,15^ and Southeast Asia^16^, and in undifferentiated fevers in Southeast Asia^17,18^. Reduction in antibiotic use in patients with respiratory tract infection was demonstrated after the integration of point-of-care CRP results in clinical algorithms designed for primary care in Tanzania^19^ and in Vietnam^16^. No study has investigated the potential usefulness of point-of-care biomarkers for the management of neurological disorders in tropical settings.

This study is a post-hoc retrospective analysis aimed at exploring the diagnostic value of CRP and PCT determination in discriminating invasive bacterial infections from other etiologies in the sera of 351 patients consecutively enrolled with neurological disorders at the rural hospital of Mosango, Democratic Republic of Congo (DRC). We assessed the diagnostic performance of CRP and PCT using the cutoff values that are provided in currently available point-of-care assays. Secondary objectives were to assess to which extent the results of CRP and/or PCT, if they had been available at initial assessment, could have modified the case management and to evaluate the prognostic value of both biomarkers for an adverse (or favorable) outcome.

## Methods

### Study participants and setting

This diagnostic accuracy study determined retrospectively the concentration of CRP and PCT in well-characterized archived clinical samples obtained from an etiological study on neurological disorders at the “Hôpital Général de Réference” (HGR) of Mosango, province of Kwilu (ex-Bandundu), Democratic Republic of Congo. The rationale, objective, design, study population and setting of this etiological study conducted within the NIDIAG project (“Better Diagnosis for Neglected Infectious Diseases”; www.nidiag.org) have been described in detail previously^20^. Briefly, children older than five years and adults who were admitted for ongoing neurological disorders were consecutively enrolled from 2012 to 2015 and prospectively followed-up for at least 3 months. Entry criteria were any of the following neurological symptoms or syndromes: altered state of consciousness; change in sleep pattern; cognitive decline; changes in personality/behavior; recent (<2 weeks) epileptic seizure; recent, severe and progressive headache; meningism; new onset cranial nerve lesion; new onset sensory-motor focal deficit; new onset gait/walking disorders. There were no exclusion criteria except non-consent by patients or legal guardians. Participants underwent a systematic and standardized workup, including a set of first-line laboratory tests in blood/serum (white blood cell [WBC] count, basic biochemistry), reference and study diagnostic assays and a microscopic examination of cerebrospinal fluid (CSF) if not contra-indicated. Advanced diagnostic methods such as neuro-imaging were not available at the study site. Several “severe and treatable” infections of the central nervous system (CNS), considered as priority conditions, were searched systematically with reference methods (most of the time later on in reference laboratories in Kinshasa, DRC or Antwerp, Belgium). Other diagnoses were made clinically following pre-established case definitions elaborated with the help of an expert in tropical neurology. A panel of clinicians expert in neurology and infectious diseases reviewed all case files in 2016 - when all additional reference test results were available - for final diagnostic ascertainment^20^.

### Study procedures

To assess the diagnostic value of CRP and PCT (index tests) in the patients enrolled in the etiological study, we grouped all study subjects within one of five clinically relevant disease categories, based on their final diagnosis as ascertained by the review panel: (1) confirmed invasive bacterial infections requiring immediate antibiotherapy (bacterial meningitis documented by CSF culture; bacteremia documented by blood culture and severe pneumonia confirmed by chest X-ray); (2) confirmed bacterial infections other than those defined here above (spinal and CNS tuberculosis, tetanus, neurosyphilis); (3) confirmed non-bacterial infections (both uncomplicated and severe malaria, human African trypanosomiasis, HIV-related opportunistic infections); (4) non-specified presumptive infections (unspecified meningoencephalitis, respiratory tract infection other than radiological pneumonia, undifferentiated febrile illness); and (5) non-communicable diseases (Table 1). This case classification was done independently and blinded to the CRP and PCT results.

**Table 1:** Median and interquartile range of C-reactive protein (n = 313) and procalcitonin (n = 317) results per diagnosis and per group of diagnoses in patients admitted for neurological disorders in the rural hospital of Mosango, Democratic Republic of Congo.

	C-reactive protein mg/L, median (IQR)	Procalcitonin µg/L, median (IQR)
**Confirmed invasive bacterial infections (n = 19)**	**233.9 (148.9–347.5)**	**10.9 (4.0–28.9)**
Confirmed bacterial meningitis (n = 13)	221.4 (142.5–346.8)	11.34 (5.67–22.52)
Bacteremia (n = 4)	304.9 (110.7–429.1)	36.91 (2.55–428.35)
Radiological pneumonia (n = 2)	160.2 (86.5–233.9)	0.52 (0.09–0.95)
**Other confirmed bacterial infections (n = 17)**	**36.4 (18.7–85.1)**	**0.09 (0.06–1.26)**
Tuberculosis of CNS (n = 4) or spine (n = 9)	55.6 (20.3–147.9)	0.19 (0.07–1.38)
Others (n = 4)*	22.2 (10.6–31.1)	0.07 (0.05–0.09)
**Confirmed non-bacterial infections (n = 25)**	**15.2 (2.5–48.1)**	**0.07 (0.05–1.6)**
Severe or uncomplicated malaria (n = 9)	34.0 (2.5–64.2)	0.41 (0.07–6.16)
HIV and related opportunistic infections (n = 7)	24.9 (13.3–41.2)	0.10 (0.07–0.15)
Second stage HAT (n = 9)	7.6 (2.5–40.7)	0.06 (0.03–0.07)
**Non-specified presumptive infections (n = 48)**	**16.2 (2.5–57.9)**	**0.07 (0.04–0.44)**
Unspecified meningoencephalitis (n = 18)	55.8 (22.8–185.6)	0.18 (0.05–0.72)
Respiratory tract infection (n = 14)	3.8 (2.5–26.4)	0.06 (0.03–0.12)
Undifferentiated febrile illness (n = 16)	2.5 (2.5–25.3)	0.06 (0.03–0.07)
**Non-communicable diseases (n = 204)**	**2.5 (2.5–6.7)**	**0.05 (0.03–0.07)**
Epilepsy (n = 55)	2.5 (2.5–2.5)	0.05 (0.03–0.06)
Anxiety-depression (n = 48)	2.5 (2.5–2.5)	0.05 (0.03–0.06)
Unspecified myelo-radiculo-neuropathy (n = 35)	2.5 (2.5–9.8)	0.05 (0.03–0.08)
Cerebrovascular accident (n = 21)	6.8 (2.5–23.2)	0.06 (0.04–0.13)
Degenerative neurological diseases (n = 17)	5.3 (2.5–7.1)	0.04 (0.03–0.07)
Metabolic diseases (n = 6)	2.5 (2.5–164.2)	0.06 (0.03–0.23)
Others (n = 22)	2.5 (2.5–9.1)	0.05 (0.03–0.23

Note: IQR denotes interquartile range; CNS central nervous system; HIV human immunodeficiency virus; HAT human African trypanosomiasis.

*Other bacterial diagnoses were tetanus (n = 3) and neurosyphilis (n = 1).

The index tests, CRP and PCT, were performed on all archived serum samples from the NIDIAG neurological study for which adequate volume was available. There were no exclusion criteria for this post-hoc analysis. After on-site processing, each serum sample had been divided into two aliquots and stored in liquid nitrogen. The samples had been shipped on dry ice to the Institute of Tropical Medicine (ITM), Antwerp, Belgium, between May 2013 and June 2015 and were stored in the biobank at −80 °C. Processing of the index tests on the preserved samples took place in April-May 2018. The laboratory technicians who performed the index tests were blinded to all other test results and to the final diagnosis of the patients. Vitros Chemistry Products CRP Slides (Ortho-Clinical Diagnostics, Rochester, NY) on Vitros 5600 Integrated System (Ortho-Clinical Diagnostics) was used for CRP determination and BRAHMS PCT-sensitive Kryptor (Thermo Fisher Scientific, BRAHMS GmbH, Hennigsdorf, Germany) on BRAHMS Kryptor Compact PLUS (Thermo Fisher Scientific, BRAHMS GmbH) for PCT determination, according to the instructions of the manufacturer. These assays have a limit of detection of 5 mg/L for CRP and of 0.075 µg/L for PCT. Reference values are set at <10 mg/L for CRP and <0.1 µg/L for PCT by the manufacturers.

In the immunochromatographic assays currently available, the lowest threshold for CRP is 10 mg/L and for PCT 0.5 µg/L. For febrile illness, antibiotic treatment is usually not recommended below these cutoffs, although some experts suggest for PCT a safer threshold of 0.25 µg/L below which antibiotics could be withheld. There is no universally accepted consensus regarding the “high” cutoff values above which antibiotic use is recommended, and not all immunochromatographic assays provide higher cutoffs. In older children and adults, a threshold of 80 to 100 mg/L is usually accepted for CRP and of 0.5 to 2 µg/L for PCT. No guidance exists for biomarker use in patients with neurological disorders.

### Data analysis and reporting

For the primary endpoint of diagnostic accuracy, median and interquartile range (IQR) of CRP and PCT concentrations were calculated for each diagnosis, and boxplots were generated for each disease category. They were compared with the WBC count determined in blood, which had been performed on site, but was missing in a subset of cases (n = 73) due to a temporary technical problem. For values of CRP and PCT below the limit of detection, the values for calculation were artificially set at 2.5 mg/L and 0.01 µg/L. Receiver operating characteristic (ROC) curves were obtained to illustrate the diagnostic ability of each biomarker to distinguish between confirmed invasive bacterial infections and other diagnoses. Areas under the curve and Youden indices were determined for CRP, PCT, and blood WBC count. The Youden index provides the “optimal” cutoff value that misclassifies the minimum number of subjects in a study population (corresponding to the point closest to the upper left corner of the ROC graph) if an equal weight is given to false positive and false negative results.

We also explored the diagnostic performances of the biomarkers at cutoff values that are usually reported in the literature as helpful to exclude or confirm invasive bacterial infections and/or are reported by diagnostics manufacturers. All results of this diagnostic accuracy study are reported according to the 2015 Standards for the Reporting of Diagnostic Accuracy Studies^21^ (STARD, checklist available as supplemental material).

For the secondary endpoints, we explored the potential impact of biomarker results on initial clinical management by critically reassessing the etiology and outcome of all “discrepant” cases, i.e. those who were administered empirical antibiotics but had normal biomarker concentrations and those clinically classified as non-infectious who had high biomarker values. Second, we determined the association of different CRP and PCT concentrations with death in bivariate and multivariable analysis; for the latter analysis, the independent clinical predictors of death identified in our previous study (neck stiffness; fever reported/documented; cachexia defined as body mass index <20; and altered consciousness defined as Glasgow Coma Scale <15)^20^ were entered in the logistic regression model together with both CRP and PCT. Of note, we did not enter blood WBC count in the model because this information was not available for the complete study population. SPSS statistics 25 and R were used for the statistical analyses. P-value of 0.05 was set for significance.

### Ethical aspects

The study protocol and the informed consent forms were approved by the Institutional Review Board of the ITM and by the Ethical Committees of the University of Antwerp, Belgium (reference 11/50/400; 2012), and of the Public Health School of Kinshasa, DRC (reference ESP/CE/016/2012). The study was performed in accordance with the standards of the Good Clinical (Laboratory) Practices guidelines and other relevant regulations, under supervision of the ITM Clinical Trials and Tropical Laboratory Units. The study was registered at clinicaltrials.gov under the identifier NCT01589289. Participants, or their legal guardians for those minor of age (<18 years) or whose neurological condition did not allow for adequate decision, were informed at recruitment and consented to future use of their stored clinical samples for further diagnostic accuracy research on the diagnosis of infectious diseases.

## Results

In total, 351 children >5 years and adults admitted for neurological disorders had been enrolled in the etiological study. Of 164 patients referred by primary care facilities, 90 (54.9%) mentioned having received antibiotics (representing 25.6% of the whole cohort). Invasive bacterial infection was confirmed in 20 of 351 participants (pre-test probability: 5.7%).

**Figure 1:**
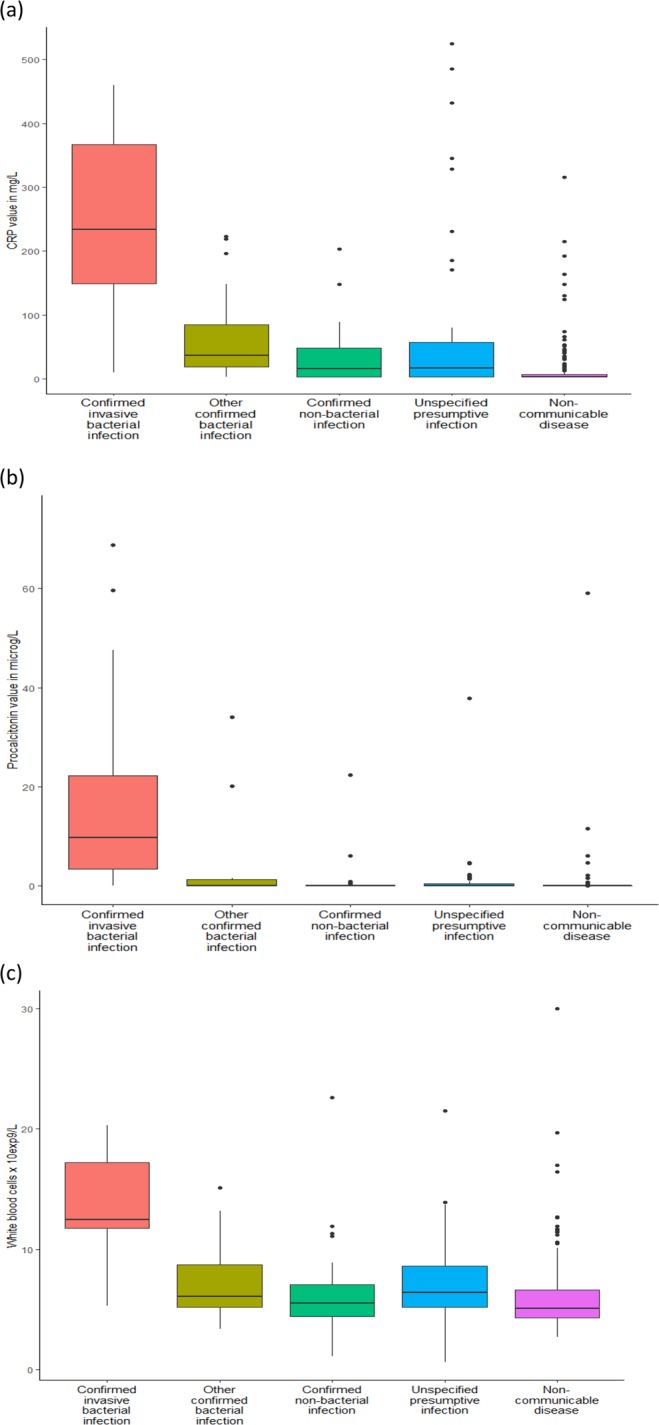
(**a**–**c**) Boxplots of C-reactive protein (CRP) results (n = 313), procalcitonin (PCT) results (n = 317) and white blood cell (WBC) count (n = 278) according to disease category. Note (Fig. 1b): two patients with very high procalcitonin levels (>100 microg/L; one in the category of confirmed invasive bacterial infection [bacteremia] and one in the category of confirmed non-bacterial infection [cerebral malaria]) are not included in graph 1(b) because of the Y axis scale.

Determination of CRP concentration could be performed in 313 (89.2%) and of PCT in 317 (90.3%) archived serum samples. The concentrations of both biomarkers are shown per diagnosis in Table 1 and per category of diagnoses in the boxplots (Fig. 1), together with the results of blood WBC count (which, for technical reasons, could only be performed in 278 participants during the prospective etiological study, see Methods). In patients with invasive bacterial infection (n = 19 for whom sufficient serum was available), the CRP and PCT concentrations were very high: median CRP 233.9 mg/L; range: 10.2–459.6 mg/L and median PCT 10.9 µg/L; range: 0.03–787.9 µg/L [results checked]), especially in cases of bacterial meningitis or bacteremia. The group of patients with a final diagnosis of non-communicable disease had very low concentrations of CRP and PCT. For the other three disease categories (other confirmed bacterial infections, confirmed non-bacterial infections and non-specified presumptive infections), median concentrations of biomarkers were intermediate, but largely below those of the group of invasive bacterial infection.

**Figure 2:**
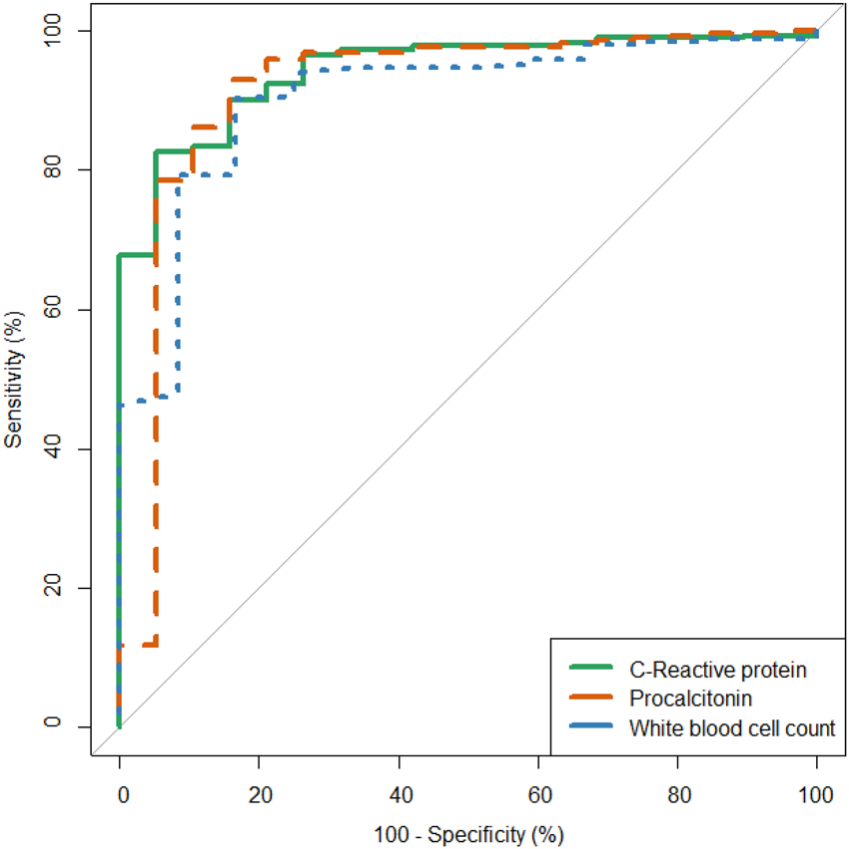
Receiver operating characteristic (ROC) curves of C-reactive protein (n = 313), procalcitonin (n = 317) and white blood cell count (n = 278) for the diagnosis of invasive bacterial infection. Note (Fig. 2): Areas under the curve were 94.3% for C-reactive protein (CRP), 91.7% for procalcitonin (PCT), and 90.5% for white blood cell (WBC) count in blood (no statistically significant difference: WBC count versus CRP *P* = 0.4; PCT versus CRP *P* = 0.6; WBC count versus PCT *P* = 0.9).

Receiver operating characteristic (ROC) curves were generated to visualize the performance of CRP, PCT, and blood WBC count for discriminating invasive bacterial infections from all the other diagnoses (Fig. 2). The areas under the ROC curve were not statistically different: 94.3% for CRP, 91.7% for PCT, and 90.5% for blood WBC count. The Youden indices (cutoff values giving the minimum number of misclassifications in the study) were calculated at 34.1 mg/L for CRP, 0.94 µg/L for PCT, and 11,150/µL for blood WBC count.

The CRP concentration was below the threshold of 10 mg/L (normal value for all reference and immunochromatographic assays) in 198/313 study patients (63.3%), and PCT was below 0.1 µg/L in 240/317 participants (75.7%). As shown in Table 2, CRP concentration was <10 mg/L in no single case of invasive bacterial infection, but PCT values were <0.1, <0.25 and <0.5 µg/L (see rationale for these cutoffs under Methods) in two, three and three such cases respectively (0.03, 0.09 and 0.23 µg/L). PCT concentration below 0.5 µg/L (lowest limit of detection in currently available immunochromatographic assays) did not decrease therefore the post-test probability of invasive bacterial infection below 1% (Table 2).

**Table 2:** Diagnostic performance of selected clinical features and values of white blood cell count and study biomarkers (C-reactive protein and procalcitonin) for the diagnosis of confirmed invasive bacterial infection in patients admitted for neurological disorders in the rural hospital of Mosango (pre-test probability: 5.7% [20/351]).

	Sensitivity	Specificity	Positive likelihood ratio	Negative likelihood ratio	Post-test probability if present/positive	Post-test probability if absent/negative
Clinical features						
Fever (reported or documented)	15/20 (75%)	245/331 (74%)	2.9	0.35	14.9	2
Glasgow Coma Scale <15	16/20 (80%)	276/331 (83.4%)	4.8	0.21	22.5	1.4
Neck stiffness	6/20 (30%)	234/331 (70.7%)	1.0	0.98	5.8	5.6
White blood cell count in blood						
>10,000/µL	10/12 (83.3%)	233/266 (87.6%)	6.7	0.15	23.2	0.8
>11,150/µL (YI)	10/12 (83.3%)	240/266 (90.2%)	8.5	0.12	27.8	0.8
C-reactive protein						
>10 mg/L	19/19 (100%)	198/294 (67.3%)	3.1	—	16.5	0
>20 mg/L	18/19 (94.7%)	221/294 (75.1%)	3.8	0.26	19.8	0.5
>34 mg/L (YI)	18/19 (94.7%)	243/294 (82.7%)	5.5	0.18	26.1	0.4
>40 mg/L	16/19 (84.2%)	245/294 (83.3%)	5.0	0.20	24.6	1.2
>80 mg/L	15/19 (78.9%)	270/294 (91.8%)	9.6	0.10	38.5	1.5
Procalcitonin						
>0.1 µg/L	17/19 (89.4%)	238/298 (79.9%)	4.4	0.22	22.1	0.8
>0.25 µg/L	16/19 (84.2%)	259/298 (86.9%)	4.8	0.16	29.0	1.1
>0.5 µg/L	16/19 (84.2%)	267/298 (89.6%)	8.1	0.12	34.8	1.1
>0.94 µg/L (YI)	16/19 (84.2%)	277/298 (93.0%)	12.0	0.08	43.2	1.1
>2 µg/L	15/19 (78.9%)	284/298 (95.3%)	16.8	0.06	51.7	1.4
>5 µg/L	13/19 (68.4%)	289/298 (97.0%)	22.8	0.04	59.1	1.4
>10 µg/L	10/19 (52.6%)	291/298 (97.7%)	22.9	0.04	59.0	3

Note: YI denotes Youden index.

Concentration of CRP >10 ml/L and PCT >0.1 µg/L had moderate positive likelihood ratios (LRs: 3.1 and 4.4 respectively) for invasive bacterial infection and increased its probability from a pre-test of 5.7% to a post-test of above 15% (Table 2). Higher cutoffs of 80 mg/L for CRP and 0.5 µg/L for PCT gave post-test probabilities of at least 30%. Both biomarkers performed better than the classic clinical predictors, while their diagnostic value was quite similar to that of the blood WBC count.

Based on the clinical assessment by the neurologist investigator at the time of patient admission, empirical antibiotic treatment was administered (either initiated or maintained) in 108 of the total cohort of 351 patients (30.8%), including ceftriaxone (for suspected invasive bacterial infection), ampicillin/penicillin, erythromycin or doxycycline, sometimes in combination with ceftriaxone (for other suspected bacterial infections). It was given to 40 of the 198 (20.2%) patients who later turned out to have normal CRP values, and were classified to have non-specified presumptive infections (n = 18) or non-communicable diseases (n = 22). Considering these 40 patients retrospectively, immediate antibiotic treatment was, most likely, not necessary. On the other hand, as shown in Fig. 1, high CRP concentrations (>80 mg/L) were observed in a few patients with a final diagnosis of non-specified presumptive infections (n = 8) or non-communicable diseases (n = 7). Incidentally, all but two got an empirical antibiotic treatment upon initial assessment. Similar proportions were found for normal/low and high PCT values (data not shown).

When analyzing the group of neurological patients with complete follow-up data and available biomarker results (Supplemental Table 1A), a normal CRP concentration (<10 mg/L) was inversely associated with death in bivariate analysis (odds ratio [OR] 0.08 (95% confidence interval [CI] 0.03–0.24; p < 0.001). A normal PCT value (<0.1 µg/L) was also inversely associated with fatal outcome, but to a weaker extent (OR 0.21 95%CI 0.09–0.49; p < 0.001). Similar strengths of association were observed when the PCT cutoffs were set at 0.25 or at 0.5 µg/L. When both biomarkers were entered in the multivariable analysis with the clinical predictors of death that had been previously identified in the etiological study (cachexia, neck stiffness, altered consciousness and fever)^20^, only a normal CRP value remained inversely correlated with death (adjusted OR 0.17 [95%CI 0.04–0.74]; p = 0.02), while fever and neck stiffness were not retained anymore (Supplemental Table 1B).

## Discussion

The care of patients presenting with neurological disorders is extremely challenging for first-line health care workers in rural areas of Central Africa, and empirical antibiotic treatment is very often administered to cope with uncertainty. Our study showed that a normal level of CRP (<10 mg/L) made the diagnosis of invasive bacterial infection very unlikely. This was not true for normal or low PCT value, which would have missed a few such cases. On the other hand, high CRP or PCT concentrations (as conventionally defined) had positive likelihood ratios (i.e. power to rule-in invasive bacterial infections) superior to that of classic clinical features and similar to that of an elevated WBC count in blood. Also, in a sizeable proportion of patients eventually diagnosed with non-specified presumptive infections or non-communicable diseases, empirical antibiotics could likely have been withheld if biomarker results had been available at initial assessment. Finally, a normal CRP value was an independent predictor of survival in this setting.

The NIDIAG etiological study had several limitations that have been summarized in previous publications^20,22^. In a subgroup of patients who were most often considered to suffer from non-infectious diseases, there was uncertainty about final clinical diagnoses, mainly because of the lack of diagnostic imaging. In addition, no specific viral screening was performed, except for herpes meningoencephalitis which was not observed. Also, our findings were limited to older children and adults and did not apply for children <5 years who have a different and better studied spectrum of diseases. For this post-hoc analysis, the number of patients with invasive bacterial infection was small. Thus we were not able to investigate the biomarker value for individual diagnoses and to robustly compare the diagnostic performance of each index biomarker. Also, since many participants had been exposed to antibiotics before referral or at admission, it was impossible to know how the availability of biomarker results would have affected the clinical evolution. Additional limitations include the duration of storage of three to five years between clinical sampling and laboratory processing of CRP and PCT, which might have affected the observed concentrations, although both CRP and PCT have demonstrated long-term storage stability^23,24^ and, in the case of CRP, repeat freeze-thawing^25^. Also, biomarker concentration was determined only once in the serum sampled at initial presentation, so the potential added-value of serial measurements could not be assessed^12^. Moreover reference (normal) values of CRP and PCT have not been specifically studied in the general Congolese population, although there is no indication of major differences with Caucasian people. Finally, it would have been preferable to prospectively evaluate the biomarker RDTs on whole blood in the study site, since performance might be lower than that of reference assays on stored serum, but the purpose of this work was exploratory. Moreover, validation of CRP-based RDTs against reference assays has been previously performed in tropical field settings, with satisfactory results^13^.

In patients evaluated for neurological disorders in similar settings, CRP and PCT determination may contribute to discriminate invasive bacterial infections (mainly bacterial meningitis) from the numerous other infectious and non-infectious etiologies. At high concentrations, both biomarkers had a confirming power superior to that of clinical features such as fever or altered consciousness, which are notoriously unreliable for identifying these most severe conditions^26^. In this study, elevated blood WBC count alone had a similar discriminative value to that of CRP and PCT. However, this has not been consistently observed for other clinical syndromes^6^. In remote settings where laboratory assays are hardly available, RDTs that detect high concentrations of CRP or PCT could help decisions to initiate appropriate antibiotics immediately, even before referral to hospitals for additional investigations.

On the other hand, and maybe more interestingly, a normal CRP value made a diagnosis of invasive bacterial infection very unlikely in our observations and was independently associated with favorable outcome. This means that CRP testing could identify neuro-infection suspects in whom antibiotics may be safely deferred. In whatever setting where administration of antibacterial therapy is the default approach for challenging neurological syndromes, a normal CRP value might help withhold antibiotic prescription in the majority of the cases. This impact could be even larger in more peripheral health facilities where the pre-test probability of invasive bacterial infection is much lower than in a referral hospital. Even in our study setting with an experienced neurologist, the availability of CRP results at initial evaluation would have avoided antibiotic treatment in a sizeable number of patients who likely did not need it. Of note, such conclusion cannot be extended to normal or low PCT values, since some severe cases would have been “missed”. This could make the field use of PCT assays, usually regarded as less sensitive/more specific than CRP, more debatable for the clinical scenario under study (initial assessment of neurological disorders with several high-acuity etiologies).

In other (non-invasive) bacterial infections, non-bacterial infections and non-specified infections, median concentrations of CRP and PCT values were found, as expected, in the grey zone. No clear-cut recommendations regarding antibiotic use could therefore be made. For some of these conditions composing the differential diagnosis of neurological disorders, other field assays have however already been widely implemented (e.g. malaria, HIV, etc.) or are being evaluated (e.g., for HAT^27^, syphilis, glycemia, oxygen saturation, etc.) to improve individual case management. Decision tools that integrate a sound set and relevant sequence of additional assays should further support clinical decision-making when biomarker values are intermediate and therefore less contributive.

Diagnostic performance and clinical usefulness of CRP and PCT (as well as new biomarkers in development^5^) are increasingly explored in many tropical fields but in very different epidemiological and clinical scenarios (primary care facilities, hospitals, undifferentiated fever, respiratory tract infection,…). There will probably not be a one-size-fits-all biomarker cut-off for all syndromes, settings, and populations. Depending on the context, biomarkers at concentrations with high positive or negative likelihood ratios should be prioritized. Also, biomarkers in isolation should not be seen as magic bullets to circumvent the clinical evaluation. However, although imperfect, they may become an important element of the diagnostic approach of neurological disorders in tropical settings, provided that validated results are integrated into sound clinical algorithms.

In conclusion, introducing point-of-care biomarker results in rural African health facilities has the potential to help identify the subsets of patients with neurological disorders at highest risk of severe disease as well as those in whom antibiotics could be safely withheld. In this latter group, the use of CRP might be safer option than that of PCT, in addition to being much less expensive at present. Prospective studies investigating the impact of such biomarkers on clinical care are urgently needed in similar resource-constrained settings and in particular at the primary care level, where advanced diagnostic technology for neurological disorders will not be available in the foreseeable future.

## Supplementary information

Supplementary Tables 1A and 1B

## References

[1] Yansouni CP (2013). Rapid diagnostic test for neurological infections in Central Africa. Lancet Infect. Dis..

[2] van de Beek D, de Gans J, Tunkel A, Wijdicks E (2006). Community-Acquired Bacterial Meningitis in Adults. N. Engl. J. Med..

[3] Hopkins H (2017). Impact of introduction of rapid diagnostic tests for malaria on antibiotic prescribing: analysis of observational and randomised studies in public and private healthcare settings. BMJ.

[4] Simon L, Gauvin F, Amre DK, Saint-Louis P, Lacroix J (2004). Serum procalcitonin and C-reactive protein levels as markers of bacterial infection: A systematic review and meta-analysis. Clin. Infect. Dis..

[5] Kapasi AJ, Dittrich S, Gonzalez IJ, Rodwell TC (2016). Host biomarkers for distinguishing bacterial from non-bacterial causes of acute febrile illness: A comprehensive review. PLoS One.

[6] Van den Bruel A (2011). Diagnostic value of laboratory tests in identifying serious infections in febrile children: systematic review. BMJ.

[7] Cals JWL, Butler CC, Hopstaken RM, Hood K, Dinant G-J (2009). Effect of point of care testing for C reactive protein and training in communication skills on antibiotic use in lower respiratory tract infections: cluster randomised trial. BMJ.

[8] Schuetz P (2018). Effect of procalcitonin-guided antibiotic treatment on mortality in acute respiratory infections: a patient level meta-analysis. Lancet Infect. Dis..

[9] Stocker M (2017). Procalcitonin-guided decision making for duration of antibiotic therapy in neonates with suspected early-onset sepsis: a multicentre, randomised controlled trial (NeoPIns). Lancet.

[10] Huang DT (2018). Procalcitonin-Guided Use of Antibiotics for Lower Respiratory Tract Infection. N. Engl. J. Med..

[11] Cortegiani A (2019). Procalcitonin levels in candidemia versus bacteremia: A systematic review. Crit. Care.

[12] Samsudin I, Vasikaran SD (2017). Clinical utility and measurement of procalcitonin. Clin. Biochem. Rev..

[13] Phommasone K (2016). Accuracy of commercially available c- reactive protein rapid tests in the context of undifferentiated fevers in rural Laos. BMC Infect. Dis..

[14] Diez-Padrisa N (2010). Procalcitonin and C-reactive protein for invasive bacterial pneumonia diagnosis among children in Mozambique, a malaria-endemic area. PLoS One.

[15] Erdman LK (2015). Biomarkers of host response predict primary end-point radiological pneumonia in Tanzanian children with clinical pneumonia: A prospective cohort study. PLoS One.

[16] Do NTT (2016). Point-of-care C-reactive protein testing to reduce inappropriate use of antibiotics for non-severe acute respiratory infections in Vietnamese primary health care: a randomised controlled trial. Lancet Glob. Health.

[17] Lubell Y (2015). Performance of C-reactive protein and procalcitonin to distinguish viral from bacterial and malarial causes of fever in Southeast Asia. BMC Infect. Dis..

[18] Wangrangsimakul T (2018). Causes of acute undifferentiated fever and the utility of biomarkers in Chiangrai, northern Thailand. PLoS Negl. Trop. Dis..

[19] Keitel K (2017). A novel electronic algorithm using host biomarker point-of-care tests for the management of febrile illnesses in Tanzanian children (e-POCT): A randomized, controlled non-inferiority trial. PLoS Med..

[20] Mukendi, D. *et al*. Clinical spectrum, etiology, and outcome of neurological disorders in the rural hospital of Mosango, the Democratic Republic of Congo. *Am. J. Trop. Med. Hyg*. **97** (2017). 10.4269/ajtmh.17-0375 PMC5817781 28820708

[21] Bossuyt PM (2015). STARD 2015: An updated list of essential items for reporting diagnostic accuracy studies. Clin. Chem..

[22] Mukendi D (2018). Where there is no brain imaging: Safety and diagnostic value of lumbar puncture in patients with neurological disorders in a rural hospital of Central Africa. J. Neurol. Sci..

[23] Schuetz P, Christ-Crain M, Huber AR, Müller B (2010). Long-term stability of procalcitonin in frozen samples and comparison of Kryptor® and VIDAS® automated immunoassays. Clin. Biochem..

[24] Doumatey A, Zhou J, Adeyemo A, Rotimi C (2014). Hig sensitivity C-reactive protein (Hs-CRP) remains highly stable in long-term archived human serum. Clin. Biochem..

[25] Macy EM, Hayes TE, Tracy RP (1997). Variability in the measurement of C-reactive protein in healthy subjects: Implications for reference intervals and epidemiological applications. Clin. Chem..

[26] Van den Bruel A, Haj-Hassan T, Thompson M, Buntinx F, Mant D (2010). Diagnostic value of clinical features at presentation to identify serious infection in children in developed countries: a systematic review. Lancet.

[27] Boelaert, M. *et al*. A phase III diagnostic accuracy study of a Rapid Diagnostic Test for diagnosis of second-stage human African trypanosomiasis in the Democratic Republic of the Congo. *Ebio Medicine*, 10.1016/j.ebiom.2017.10.032 (2017). 10.1016/j.ebiom.2017.10.032 PMC5828295 29246478

