# Supplementary material for: Potential usefulness of C-reactive protein and procalcitonin determination in patients admitted for neurological disorders in rural Democratic Republic of Congo
Source: Sci Rep. 2019 Oct 29;9:15505. doi: 10.1038/s41598-019-51925-z (PMC6820716; doi:10.1038/s41598-019-51925-z)
Supplement: Supplementary file 1 — Supplementary Tables 1A and 1B [file 41598_2019_51925_MOESM1_ESM.pdf]

Potential usefulness of C-reactive protein and procalcitonin determination in patients admitted for neurological disorders in rural Democratic Republic of Congo

Emmanuel Bottieau[1]*, Deby Mukendi[2,3], Jean-Roger Lilo Kalo[2] , Pascal Lutumba[2,3] , Barbara Barbé[1], Kadrie Ramadan[1], Marjan Van Esbroeck[1], Jan Jacobs[1,4], Cedric P Yansouni[5], François Chappuis[6], Marleen Boelaert[7], Andrea S Winkler[8,9], Kristien Verdonck[7]

Supplemental Table 1A: Bivariate analysis of the association between C-reactive protein and procalcitonin concentrations at selected values and fatal outcome in patients admitted for neurological disorders and with complete follow-up data in the rural hospital of Mosango

Biomarkers values	Fatal outcome	Survival	*P*	Odd ratio (CI95%)
**C-reactive protein**	**N=26**	**N= 251**		
Median value, in mg/L (IQR)	55.1 (19.0-192.9)	2.5 (2.5-16.4)	< 0.001	
< 10 mg/L	4 (15.4%)	175 (69.7%)	< 0.001	0.08 (0.03-0.24)
> 80 mg/L	10 (38.5%)	21 (8.4%)	< 0.001	6.8 (2.8-17.0)
**Procalcitonin**	**N=26**	**N=254**		
Median value, in µg/L (IQR)	0.13 (0.07-11.4)	0.05 (0.04-0.08)	< 0.001	
< 0.1 µg/L	12 (46.2%)	204 (80.0%)	< 0.001	0.21 (0.09-0.49)
< 0.25 µg/L	15 (57.7%)	221 (87.7%)	< 0.001	0.21 (0.09-0.49)
< 0.5 µg/L	16 (61.5%)	226 (89.0%)	< 0.001	0.20 (0.08-0.48)
> 2 µg/L	8 (30.8%)	20 (7.8%)	< 0.001	5.2 (2.0-13.5)
> 10 µg/L	7 (26.9%)	9 (3.5%)	< 0.001	10.1 (3.4-30.0)

Note: IQR denotes interquartile range; CI confidence interval

Supplemental Table 1B: Multivariable analysis of the association between clinical predictors, C-reactive protein, and procalcitonin results, and fatal outcome in patients admitted for neurological disorders and with complete follow-up data in the rural hospital of Mosango (n=277)

Variables	Adjusted odd ratios (95%CI)	*P*
**Clinical features**		
Altered consciousness (Glasgow < 15)	5.43 (1.79-16.52)	0.003
Neck stiffness	1.39 (0.50-3.88)	0.53
Cachexia (body mass index < 20)	6.51 (2.03-20.92)	0.002
Fever (reported/documented)	2.66 (0.88-8.02)	0.08
**Laboratory features**		
C-reactive protein < 10 mg/L	0.17 (0.04-0.74)	0.02
Procalcitonin < 0.1 µg/L	1.78 (0.55-5.80)	0.33

Note: CI denotes confidence interval
